# Complicating Acute Myocardial Infarction. Current Status and Unresolved Targets for Subsequent Research

**DOI:** 10.3390/jcm10245904

**Published:** 2021-12-16

**Authors:** Jaroslaw Zalewski, Karol Nowak, Patrycja Furczynska, Magdalena Zalewska

**Affiliations:** 1 Department of Coronary Disease and Heart Failure, Jagiellonian University Medical College, 80 Pradnicka Street, 31-202 Krakow, Poland; k.nowak.uj@gmail.com (K.N.); f.patrycja@wp.pl (P.F.); 2 Department of Emergency Medicine, Faculty of Health Science, Jagiellonian University Medical College, Michalowskiego 12 Street, 31-126 Krakow, Poland; magdalena.zalewska@uj.edu.pl

**Keywords:** myocardial infarction, cardiogenic shock, ventricular septal rupture, ventricular free wall rupture, mitral regurgitation, mechanical circulatory support, left ventricular assist device

## Abstract

Mechanical reperfusion with primary angioplasty, as the treatment of choice in acute myocardial infarction (MI), is associated not only with a high percentage of full epicardial and tissue reperfusion but also with a very good immediate and long-term clinical outcome. However, the Achilles heel of MI treatment is its ensemble of complications, such as cardiogenic shock due to severe systolic and/or diastolic dysfunction or MI mechanical complications, including perforation of the left ventricular free wall, papillary muscle rupture with acute mitral regurgitation and ventricular septal rupture. They are associated with an increased or, sometimes, with an extremely high mortality rate, determining the overall mortality in an MI patient population. In this review we summarize the mechanisms of MI complications, current therapeutic management and alternative directions for overcoming their devastating consequences. Moreover, we have sought to indicate gaps in the evidence on current treatments as the potential targets for further clinical research. From the perspective of mortality trends that are not improving, the forthcoming therapeutic management of complicated MI will require an individualized and novel approach based on their thorough pathobiology.

## 1. Introduction

Worldwide, coronary artery disease (CAD) is the single most frequent cause of death. Some 550 thousand deaths were attributed to CAD across European Union countries in 2017, accounting for 12% of all deaths [1]. The in-hospital mortality of unselected ST-elevation myocardial infarction (MI) patients in the national registries of the ESC countries varies between 4% and 12% and is mainly driven by acute heart failure or cardiogenic shock (CS) due to severe left ventricular (LV) systolic dysfunction [2].

Recent summaries indicate varying trends with a decreased, stable, or even increased incidence of CS ranging from 3% to 15% in acute MI patients within the last two decades [3,4]. Between 2005 and 2017 in Denmark, out of 101,834 acute MI patients, 7% had CS. In the study period, the use of coronary angiography increased from 48% to 71%, while, the use of LV assist devices increased from 1% to 10% and of norepinephrine from 30% to 70%. In contrast, the use of an intra-aortic balloon pump (IABP) decreased from 14% to 1% and of dopamine from 34% vs. 20%. Over time, the mortality in the CS population has remained high, but decreased, from 68% in 2005 to 57% in 2017 (*p* < 0.001 for temporal change) [5]. Between 2003 and 2010, from the United States Nationwide Inpatient Sample databases and similar EU databases, including 1,990,486 patients aged ≥40 years with ST-elevation MI, 7.9% had CS. Over the 8-year period, the use of early mechanical revascularization increased from 30.4% to 50.7% and of IABP from 44.8% to 53.7%. Simultaneously, risk-adjusted in-hospital mortality decreased significantly, from 44.6% to 33.8% (*p* < 0.001), whereas the average total hospital cost increased, from about $36,000 to $46,000 (*p* < 0.001) [6].

Among more than 9 million MI patients in the United States, included in National Inpatient Sample database for the years 2003–2015, mechanical complications were identified in 0.12% of hospitalizations, with no changes in trends over time in either the STEMI or NSTEMI populations [7]. The rates of in-hospital mortality in patients with mechanical complications were 42.4%, after STEMI, and 18.0%, after NSTEMI, and they continued to be high during the study period. In turn, in patients aged ≥75 years, the incidence of mechanical complications decreased between 1988 and 2008, from 11.1% to 4.3%, and, here, in parallel with the increasing use of reperfusion therapy, particularly primary angioplasty. Nevertheless, there were no significant changes in patient hospital fatality over the 20-year period, which was 87.1% in 1988 and 82.4% in 2008 [8].

Following MI, every ninth CS is caused by a mechanical complication [9]. Although, the incidence of mechanical complications has declined in parallel with the progressive use of reperfusion therapy, irrespective of the study period, about one fourth of deaths after MI are still caused by mechanical complications, including 15% following a perforation of the LV free wall, 5% after papillary muscle rupture or its dysfunction with acute mitral regurgitation and 5% after ventricular septal rupture [10,11].

From the perspective of not-improving mortality trends, the development of new, effective therapies, together with individualized algorithms dedicated to complicating acute MI, may be associated with substantial progress in the management of this civilization-wide disease. The goal of this review is to summarize the mechanisms of MI complications, and to indicate gaps in the evidence on the current treatment as the targets for further research on existing and alternative solutions for overcoming the devastating consequences of complicating acute MI.

## 2. Mechanisms of Dysfunction in Complicating MI

**Figure 1:**
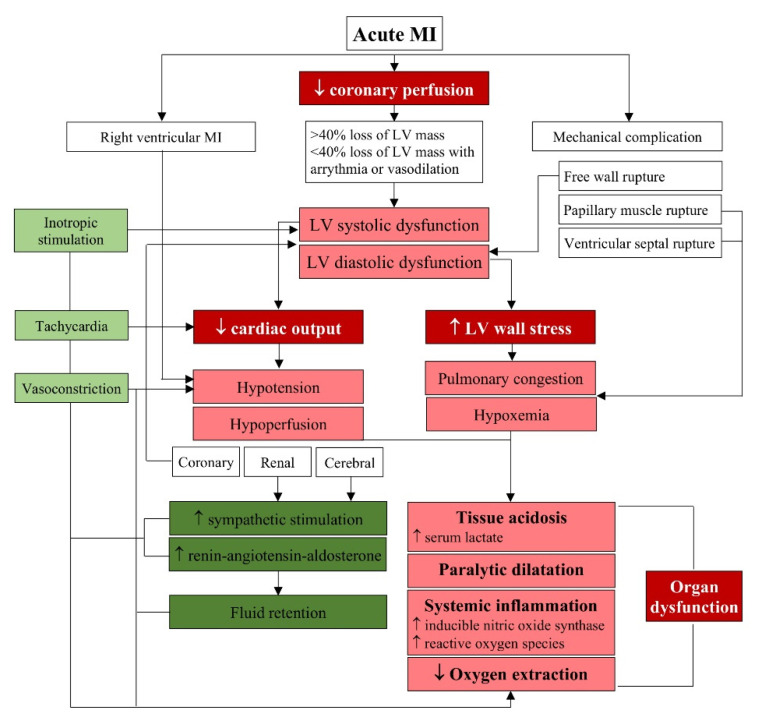
Pathophysiology of cardiogenic shock. The primary cause of cardiogenic shock is severe myocardial injury and cardiac dysfunction leading to a supply–demand imbalance in the peripheral end-organs. Early endogenous compensatory mechanisms are activated to prevent the irreversible consequences of hypoperfusion and hypoxemia. Abbreviations: MI myocardial infarction, LV: left ventricular.

The severe depression of LV contractility following MI leads to its systolic dysfunction with reduced stroke volume and/or diastolic dysfunction with increased LV wall stress and pulmonary congestion (Figure 1). Thus, the predominant clinical symptoms of CS include peripheral hypoperfusion of the central nervous system with impaired consciousness, kidneys with oliguria/anuria of less than 0.5 mL/kg/h and skin pale and cold. An unspecific symptom of a CS but one helpful in its initial diagnosis is the accompanying hypotension of systolic blood pressure of less than 90 mmHg for at least 30 min or with the need for pharmacologic support, while the parameter confirming the cardiogenic source of peripheral hypoperfusion is a cardiac index lower than 2.2 L/min/m^2^ and a pulmonary wedge pressure higher (PCWP) than 15 mmHg [12,13,14]. Tissue hypoperfusion in patients with CS leads to tissue acidosis. To limit the consequences of hypoperfusion, early endogenous compensatory mechanisms, including tachycardia, inotropic stimulation and vasoconstriction are activated. Tachycardia is energy inefficient; if the stroke volume impairment is too deep and the compensatory tachycardia is inadequate, the shock symptoms worsen. Inotropic stimulation is helpful in the initial normalization of the stroke volume; however, further intracellular cardiac myocyte concentration of secondary messengers is reduced and the effect of adrenergic stimulation becomes attenuated. Nevertheless, despite adrenergic stimulation, myocardial strain of necrotic or stunned myocardium in shock patients remains substantially reduced. Thus, a cardiac power output as a simultaneous measure of both flow and pressure domains of cardiovascular system and calculated as cardiac output × mean arterial pressure has proven to be the strongest independent hemodynamic correlate of in-hospital mortality [15].

Patients with symptoms of acute right ventricular (RV) failure are characterized by hypotension and filled jugular veins. RV systolic dysfunction following inferior wall MI involving the right ventricle, exacerbates LV shock symptoms. If the RV function is not impaired, a mismatch between the ventricles leads to an increase in PCWP and an increase in LV end-diastolic pressure. Too high a mismatch between a properly functioning right and significantly impaired left ventricle may be associated with leakage into the alveoli and the formation of pulmonary edema [16,17,18].

Among 10,004 patients with acute coronary syndrome or heart failure, adjusted hospital mortality in subjects with isolated hypoperfusion was higher than in patients with isolated hypotension (17.2% vs. 9.3%, *p* = 0.02) and not significantly different from patients with both hypotension and hypoperfusion (33.8%, *p* = 0.18) [19]. Based on these observations, the experts of the Society for Cardiovascular Angiography and Interventions developed a new, clinically useful, five-stage classification of CS [20]. Patients at stage A are at risk of CS. Stage B indicates the beginning of CS with tachycardia, hypotension but without hypoperfusion. Stage C is a classic CS with hypoperfusion, a cardiac index < 2.2 L/min/m^2^ and PCWP > 15 mmHg. Stage D, as deteriorating, CS implies that the initial set of interventions chosen, including pressors and/or mechanical circulatory support (MCS), have not restored stability and adequate perfusion despite at least a 30-min observation. In the extreme E stage the patient is highly unstable and often vulnerable to cardiovascular collapse.

In patients with CS, microcirculation dysfunction is an early feature preceding the clinical symptoms of organ failure. In a pre-shock stage B arterioles and venules constrict while capillaries close in order to maintain driving pressure [21]. It was shown that, in CS, the response to reactive hyperemia is attenuated. This appears to reflect increased vasoconstriction and an impaired capacity for vasodilation. Additionally, decreased erythrocyte deformability but not neutrophil–endothelial cell interactions are important in limiting systemic microvascular flow [22]. In turn, vasoconstriction facilitates blood mobilization from the viscera, increasing preload. Moreover, the reduction of renal blood flow stimulates the sympathetic system, activates the renin–angiotensin–aldosterone axis, the latter of which enhances fluid retention. Usually, the decrease in renal perfusion during CS is relatively greater than the reduction in cardiac output, most likely due to increased central venous pressure. From stage C onward, arterioles dilate and in stages D and E they become paralyzed, which is associated with a blood pressure drop resistant to pharmacological control. Simultaneously, capillary stasis leads to microthrombosis and, in stage E, to uncontrolled endothelial permeability. As a result, unbalanced tissue hypoperfusion, starting in stage C, intensifies lactate accumulation and tissue acidosis [21]. 

There is an independent association between microcirculatory perfusion parameters expressed as the proportion of perfused capillaries or perfused capillary density and the combined clinical endpoint of all-cause death and renal replacement therapy at 30 days follow-up. Moreover, in patients with loss of hemodynamic coherence between macrocirculatory and microcirculatory perfusion parameters, the latter have dominant prognostic value [23]. On the other hand, an increase of mean arterial pressure from <60 mmHg to 60–90 mmHg did not affect microcirculation variables in CS patients with ECMO support [24].

In the ischemic microcirculation of MI patients with CS a strong and highly variable inflammatory response is detected, but it does not reach the intensity of the inflammation observed in patients with septic shock. There is an excessive production of reactive oxygen species and cytokines, the activation of the complement system, and the stimulation of neutrophils, platelets and endothelial cells [14,25]. Moreover, an extensive immune/inflammatory response, as reflected by the inflammatory markers IL-6, -7, -8 and -10, is associated with poor prognosis [26,27]. One fifth of patients with acute MI complicated by CS showed clinical signs of severe systemic inflammation, but those who were culture-positive for sepsis had twice the risk of death [28]. The prevalence of infection in CS patients is estimated at 20–45%, and respiratory tract infections are the most common [29]. In the setting of CS, hypoperfusion and congestion in the intestines can alter gut morphology, permeability and function, and, possibly, the growth and composition of gut microbiota. These changes can disrupt bowel barrier function and exacerbate systemic inflammation via microbial or endotoxin translocation into systemic circulation. Here, the most prominent factor is endotoxicity as a basic mediator of gram-negative bacteria, which also triggers the activation of both humoral and cellular systems [30]. The role and time of occurrence of inflammation need further clarification.

## 3. Pathophysiology of Mechanical Complications of MI

Before the era of fibrinolytic reperfusion ventricular septal rupture (VSR) or papillary muscle rupture (PMR) complicated 1–3% of MI, whereas free wall rupture (FWR) complicated 2–6% of MI [11,31,32]. In the era of thrombolytic reperfusion the incidence of every type of mechanical complication following MI decreased significantly, to 0.2–0.3% [11,33,34]. Moreover, using primary percutaneous coronary intervention (PCI) the incidence of VSR and FWR was further reduced [35,36,37]. In MI patients without reperfusion therapy, mechanical complications typically occur within the first two weeks after MI, peaking 3–7 days from the onset of symptom [11,33].

During the first 24 h there are relatively few neutrophils within the infarct tissue, however, coagulative necrosis is then just starting, and early ruptures occur in the infarct area with large intramural hematomas that penetrate heart tissues and dissect the LV walls. In the next days the risk of mechanical complications is associated with neutrophils infiltrating the infarct zone, where they release lytic enzymes, hastening the disintegration of necrotic myocardium. Although thrombolysis is necessary to reduce the infarct size, in some cases it may promote hemorrhagic dissection in the LV wall, accelerating the onset of its rupture.

The size of VSR ranges from millimeters to a few centimeters. Simple VSR has a discrete channel at the same level on both sides of the perforation. In contrast, a complex rupture is characterized by a large intramural hematoma and a channel of an irregular shape, penetrating necrotic tissue [38]. A septal rupture results in a left-to-right shunt, with right ventricular volume overload, increased pulmonary blood flow, and secondary volume overload of the left atrium and ventricle. As the LV systolic function deteriorates and the forward flow declines, compensatory vasoconstriction leads to increasing systemic vascular resistance, which, in turn, increases the magnitude of the left-to-right shunt. The degree of shunting is determined by the size of the septal rupture, by both pulmonary and systemic vascular resistance, and by left and right ventricular function. As the left ventricle fails and the systolic pressure declines, left-to-right shunting decreases and the fraction of the shunt diminishes.

The degree of acute mitral regurgitation (MR) following PMR depends on the dysfunction or rupture of the papillary muscle and the accompanying changes in LV geometry and/or dysfunction [39]. Even slight alterations of LV geometry due to pathology in regional contractility may contribute to an increase in the MR frequency after MI [40]. A rupture of the posterior-medial papillary muscle is observed 3–12 times more often than that of antero-lateral [41]. The antero-lateral papillary muscle is less prone to rupture due to its double blood supply from the left anterior descending artery and the left circumflex artery. In turn, the posterior-medial papillary muscle is more sensitive to ischemic injury, as its blood supply is derived only from the posterior descending artery [42]. PMR may be partial, when it affects one of the papillary muscle heads and is usually observed in the rupture of the posterior-medial muscle, or complete, which is more common in the antero-lateral muscle [43]. Partial PMR leads to MR of a different degree, whereas complete PMR causes a prolapse of both mitral valve leaflets associated with severe MR. Bouma et al. [44] showed that in-hospital mortality in patients with complete PMR was 42% while in partial PMR was more than three times lower. In MI accompanied by a PMR there is no time for the small left ventricle and the left atrium to adapt to new conditions. Therefore, the symptoms of cardiogenic shock or pulmonary edema develop rapidly.

Following FWR, pericardial tamponade, electromechanical dissociation, and finally death usually occur. In some cases, a clot adjacent to the rupture can close the leak into the pericardium and lead to the formation of a pseudoaneurysm. Based on the pathological criteria, FWR can be divided into type I, with a sudden fissure rupture in the myocardium of the time of ischemia < 24 h, type II, with an erosion site in the infarct zone showing a gradual worsening of the tear and type III rupture, associated with early LV aneurysm formation. Clinically, according to the size of FWR and the dynamics of bleeding to the pericardium, FWR can be divided into an oozing type and a blowout type [45].

## 4. Risk Factors of Complicating MI and Predictors of Clinical Outcomes

The heterogeneity in the definition of CS, patient characteristics, management strategies, and outcome measures creates many different factors independently associated with the occurrence of CS. Several methodological imperfections including bias associated with patients’ selection in observational studies, variable selection and an inadequate sample size make it difficult to assess objectively which factors are in fact initial and most important. The CS symptoms may develop before hospital admission or subsequently during hospitalization. Ay older age, an out-of-hospital cardiac arrest and STEMI were associated with both types of CS [46,47]. Pre-hospital CS was more likely in patients with a history of heart failure. Higher CRP levels, a left bundle branch block or a right bundle branch block on admission were associated with an increased risk of developing in-hospital CS. In turn modern antiplatelet or anticoagulant therapy was associated with a lower risk of in-hospital CS [46]. Acharya [47] reviewed the CS risk factors, among which the most often reported were an older age, lower systolic blood pressure and/or a higher heart rate on admission, anterior wall MI, prior MI and female sex. For short- and long-term outcomes, revascularization has if of benefit at all risk levels in CS patients. Important predictors of outcomes are also hemodynamic parameters and measures of end-organ perfusion, including lactate. An evolving concept of door-to-unloading time awaits well-controlled clinical trials.

The mechanical complications were accompanied by CS in 53.5% of STEMI patients and in 23.9% of NSTEMI subjects [7]. In STEMI patients with mechanical complication and CS, independent predictors of higher mortality were an older age, female sex, valvular heart disease, peripheral artery disease, obesity, a history of coronary artery bypass grafting (CABG), the use of a percutaneous ventricular assist device, extracorporeal membrane oxygenation (ECMO) and the use of systemic thrombolytics. In the NSTEMI population, an older age and the use of percutaneous ventricular assist device contributed to higher mortality. Lower mortality in patients with mechanical complications who developed CS was associated with a surgical repair in the STEMI and NSTEMI cohorts and with PCI in the STEMI cohort.

In STEMI patients within 12 h from the onset of the symptom female sex, a multivessel disease, the development of a collateral flow to the infarct-related artery and decreased LV ejection fraction but not time to reperfusion, the infarct location and the epicardial blood flow according to TIMI scale after PCI were the independent risk predictors of significant ischemic MR [47]. In turn, in patients who underwent a mitral valve replacement due to PMR, a low cardiac output, renal failure and treatment with ECMO were associated with in-hospital death [48,49]. Bouma et al. showed that the value of a logistic EuroSCORE index of more than 40% or a logistic EuroSCORE II index of more than 25% independently predicted death in patients following PMR surgery with an accuracy of 83–85% [44].

Morillon-Luton et al. [50] found that in patients with VSR there was a significant reduction of 1-year mortality between the eighties and nineties of the last century, most likely due to the increased prevalence of thrombolytic therapy. A similar improvement did not occur in the last decade of the study, although this was expected due to the development of catheter-based reperfusion in MI. In turn, Moreyra et al. [51] in a large-scale retrospective analysis including almost 150,000 MI patients treated between 1990 and 2007 found that, in a VSR cohort over an 18-year follow-up, the overall in-hospital mortality rate (41% in 1990–1992 and 44% in 2005–2007) and 1-year mortality rate (60% in 1990–1992 and 56% in 2005–2007) did not change significantly. After a multivariate adjustment, an increasing age and CS were the only independent predictors of mortality in VSR patients.

In the GRACE registry ST-segment elevation or depression, a left-bundle branch block, female gender, a prior stroke, a significant increase of cardiac necrotic markers, an advanced age, and tachycardia were associated with FWR following ACS [37]. In contrast, FWR was observed less frequently in patients treated with low-molecular-weight heparin and beta-blockers in the first 24 h of MI and in those with prior MI. In the registry by Yip H-K et al. [35] in a population of 1,250 Chinese patients with MI, the use of primary PCI significantly reduced the risk of FWR, but anterior wall MI was associated with a higher incidence of this complication. The 30-day and 1-year mortality rates in patients with FWR after cardiac surgery in the GUSTO registry were 47 and 53%, respectively, as compared to 94 and 97% in nonsurgical patients.

Recently, quantitative proteomics analyses of CS patients allowed the identification of a new CS4P risk assessment classifier, composed of four circulating proteins, including liver-type fatty acid-binding protein, beta-2-microglobulin, fructose-bisphosphate aldolase B and serpinG1, and associated them with multi-organ dysfunction, inflammation and immune activation. The combination of the CS4P model to existing risk scores may improve their predictive metrics and may help clinicians in the early identification of high-risk CS patients for prompt invasive procedures, including MCS [52].

## 5. Principles for the Management of Patients with Complicating MI

Percutaneous or surgical revascularization in MI patients with symptoms of CS is beneficial [53]. After 6 months, the survival rate was 63.1% in the revascularized group as compared with 50.3% in the conservative group (*p* = 0.03). Although the positive effect associated with revascularization was present only in the group of patients up to 75 years of age, it persisted for many years after intervention [53,54]. The results of the SHOCK trial and its subsequent subanalyses have provided arguments for the class IB recommendation of the European Society of Cardiology on revascularization in MI complicated by acute heart failure [55]. The results of the CULPRIT-SHOCK trial showed that in MI patients with CS and multivessel coronary disease, the 30-day composite of death or severe renal failure leading to renal replacement therapy was lower among those who only underwent PCI of the culprit lesion than it was among those who underwent multivessel PCI [56,57,58]. Despite full epicardial blood flow restoration following primary coronary angioplasty in acute MI, ischemia/reperfusion is associated with cardiac myocyte necrosis, coronary microvasculature damage and interstitial edema [59], leading to a lack of adequate tissue perfusion, referred to as the no-reflow phenomenon [60]. Even a small amount of microvascular damage can be detected by cardiovascular magnetic resonance as microvascular obstruction [61]. Its occurrence in MI patients is associated with scar formation, left ventricular remodeling and worse clinical outcomes [62,63]. It was also shown that patients with increased shock index, defined as the ratio of heart rate and systolic blood pressure, had larger microvascular obstruction and reduced major adverse cardiac event-free 12-month survival [64].

More than 90% of CS patients receive inotropes and/or vasopressors. Such vasoactive treatment restores haemodynamics while increasing myocardial oxygen consumption. Thus, the current approach, with a reliance on systematic first-line vasopressor therapy, requires further research concerning the optimization of myocardial supply/demand imbalance and organ perfusion. Additional clinical studies are expected to find optimal dose, up-titration and combination of vasopressors and inotropes, as well as details concerning how and when to wean the patients from inotropes/vasopressors. The new treatment regimens should be tested with a stepwise, dynamic, and functional approach, including the macro- and microcirculation effects of different drugs with various effects [18].

**Figure 2:**
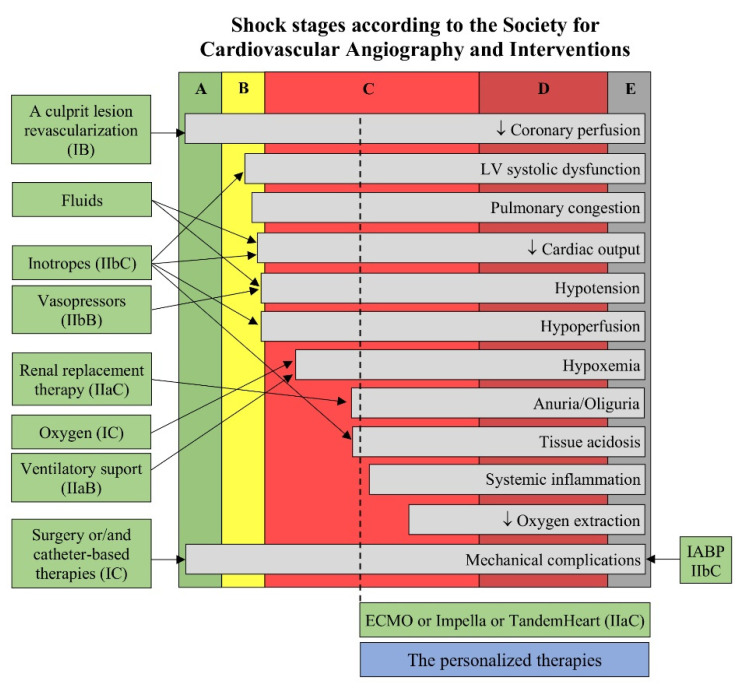
Current treatment of cardiogenic shock tailored to its stage. The available therapeutic methods with their classes of recommendation according to the European Society of Cardiology [12] have been assigned to pathological processes within the A to E stages of cardiogenic shock [20]. The personalized, innovative solutions are particularly expected in the higher stages of cardiogenic shock. Abbreviations: IABP: intra-aortic balloon pump, ECMO: extracorporeal membrane oxygenation.

Acute respiratory failure is present in almost all CS patients. About one third of them develop acute kidney injury, while more than 50% of patients present with elevated liver enzymes. If the management of a CS patient, including the administration of inotropes (class IIbC) and/or vasopressors (IIbB), revascularization of the culprit lesion (class IB), oxygen (class IC), ventilatory support (class IIaB), renal replacement therapy (class IIaC) and surgery in the case of mechanical complications (Figure 2) with hemodynamic instability (class IC) is insufficient, short-term MCS should be considered as a bridge to recovery, to decision or to bridge (class IIaC) [12]. MCS in the CS is used for the temporary replacement of an insufficient LV systolic function to cover peripheral blood demand.

## 6. Well-Established Therapeutic Approach to Mechanical Complications

So far, the optimal time window for cardiac surgery for VSR has not been established. There is evidence for immediate surgical repair of VSR, regardless of the hemodynamic status of the patient to avoid further acceleration of the CS symptoms. The argument for early VSR closure is that the septal branches of the coronary arteries, subjected to shear stress of blood flow, as well as the process of demarcation necrosis, promote expansion of VSR, which may cause hemodynamic decompensation. On the other hand, many surgeons believe that surgery should be postponed by 3–4 or even 6 weeks, until the fibrotic scar is formed in the adjacent necrotic tissue, which allows the surgeon to safely and effectively suture the edges of the rupture and to prevent VSR recanalization. Early surgery of the defect in the acute phase of MI, when the edges of the rupture are fragile, is often associated with the risk of recurrent VSR, which usually leads to lethal circulatory decompensation. However, it should be noted that delaying surgery for 3–6 weeks in a patient with signs of a significant left-to-right shunt carries the risk of rupture extension and death while waiting for surgery.

Irrespective of the optimal time window, surgical repair of postinfarction VSR is the treatment of choice (class IC) [12]. Without this treatment, 90% of patients may die within one month [34]. In the largest to date observational study, the overall 30-day mortality in VSR patients was 43% and was inversely associated with the time elapsed since VSR onset to cardiac surgery [65]. Cardiac surgery may be considered with the support of IABP (Class IIbC) [12,66] or another MCS (IIaC). Since the first surgical VSR closure in 1957, the overall mortality in this disease remains extremely high and is oscillating between 20 and 87% [67]. 

In patients with PMR, initial pharmacological treatment is aimed at afterload lowering, reducing the fraction of regurgitation, and increasing the forward stroke volume with the use of vasodilators, diuretics and IABP. In a refractory CS it is usually necessary to use multiple catecholamine infusions, and when these are insufficient short-term MSCs such as Impella [68,69], TandemHeart or ECMO may be applicable [12,70,71,72].

Since treatment pharmacologically, alone, is associated with 50% mortality rate in the first 24 h and 80% within the first week, cardiac surgery is the treatment of choice in patients with PMR [44,73,74,75]. If there is evidence for papillary muscle necrosis, or there are concerns about the progression of its ischemic damage, mitral valve replacement becomes the only effective treatment. On the other hand, if acute MR is a consequence of partial PMR and the degree of damage to the adjacent myocardial tissues is limited, repair of the mitral apparatus might be considered [43,76,77]. In the group of patients undergoing valve replacement, in whom the muscle-valve ring continuity was interrupted, the mortality rate was the highest and reached 50% [44]. Preoperative hemodynamic instability or symptoms of CS may be an indication for the use of IABP that reduces afterload and improves coronary perfusion [44]. Bouma et al. [43] showed that the use of IABP before surgery was not associated with a worse prognosis, but the fact of IABP implantation during surgery was a strong independent risk factor of subsequent in-hospital death. 

Although PMR leads rapidly to hemodynamic instability, in those patients LV ejection fraction assessed by echocardiography is usually well preserved [76]. The available studies show that only about 20% of patients with PMR had moderately impaired LV ejection fraction between 30–50%, and only one in ten had severely impaired LV ejection fraction of less than 30%. Moreover, the value of LV ejection fraction had no prognostic impact [49], most likely since well-preserved LV ejection generates enhanced the shear stress associated with a higher risk of rupture of the ischemic papillary muscle [76].

There is a discussion as to whether to perform CABG or not during PMR surgery. In one of the largest cohorts of 126 patients with PMR treated with surgery, the perioperative mortality was 27% and the 15-year survival rate was 39% [74]. Although no difference in in-hospital mortality was observed in patients following mitral valve replacement due to PMR with or without simultaneous CABG (27.3% vs. 26.4%, respectively), 64% of revascularized patients and only 23% of non-CABG patients survived 15 years (*p* < 0.001). In contrast, Russo et al. [77] analyzed the results of 54 patients with PMR treated between 1980 and 2000 and found that the reduction in in-hospital mortality from 67% to nearly 9% was associated with CABG surgery accompanying mitral valve surgery in the second decade of the analyzed period. Interestingly, patients who survived 30 days after surgery due to PMR had a similar 5-year prognosis as patients with uncomplicated MI, matched for age, sex, ejection fraction and infarct location. The study findings by Schroeter et al. [49] and Bouma et al. [44] consistently indicate that simultaneous CABG in patients with PMR surgery does not improve the hospital outcomes. It is possible that the benefits associated with the improved myocardial perfusion following CABG are reduced by the longer perioperative ischemia. Therefore, a hybrid procedure in which mitral valve surgery is preceded by PCI of an infarct-related artery is a therapeutic option to consider. Currently, there are no arguments from randomized controlled trials for or against revascularization accompanying PMR valvular surgery, nor arguments for a surgical, percutaneous or hybrid method of performing it.

**Figure 3:**
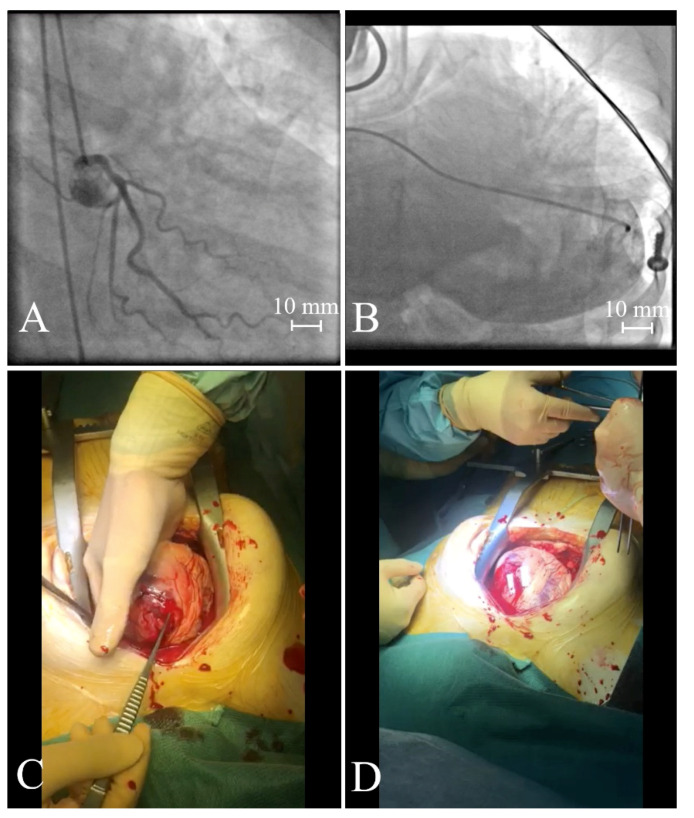
A patient with blowout type of free wall rupture. The acute ostial occlusion of left anterior descending artery (**A**). Pericardiocentesis (**B**). A blowout type of anterior wall rupture (**C**) sutured by a cardiac surgeon (**D**).

In most cases, acute FWR is associated with sudden death. The dynamics of symptoms make even in-hospital diagnosed acute FWR difficult to treat effectively. Subacute ruptures give a chance for effective treatment if they are diagnosed and transferred immediately to a tertiary center. The gold standard in the treatment of FWR is cardiac surgery, which allows for decompression of the tamponade and repair of the rupture. In the case of rapidly increasing symptoms, surgery should be preceded by pericardiocentesis. Unfortunately, the mortality rate among patients treated with surgery remains high, reaching 32% [45]. The largest to date meta-analysis by Matteucci et al. [45] included 363 patients with FWR who underwent surgery. They revealed a twice-lower operative risk in patients with oozing type rupture, as compared to the blowout type (Figure 3 and Video S1). Risk of death was 40% lower in subjects in whom FWR was treated with a sutureless technique, as compared to those undergoing sutured repair. Sutureless repair of the FWR can be performed using a collagen sponge, or pericardium patch fixed on the epicardium with glues, to cover the infarcted myocardium. In contrast, the sutured technique is defined as a repair of the FWR using sutures to close any myocardial tear or to secure a patch on the epicardium. The oozing perforations can be sewn, patched, or closed with tissue glue [78]. In survivors, sutured PMR must be monitored for early detection of leaks or for aneurysm formation.

## 7. Mechanical Circulatory Support for Complicating MI

**Figure 4:**
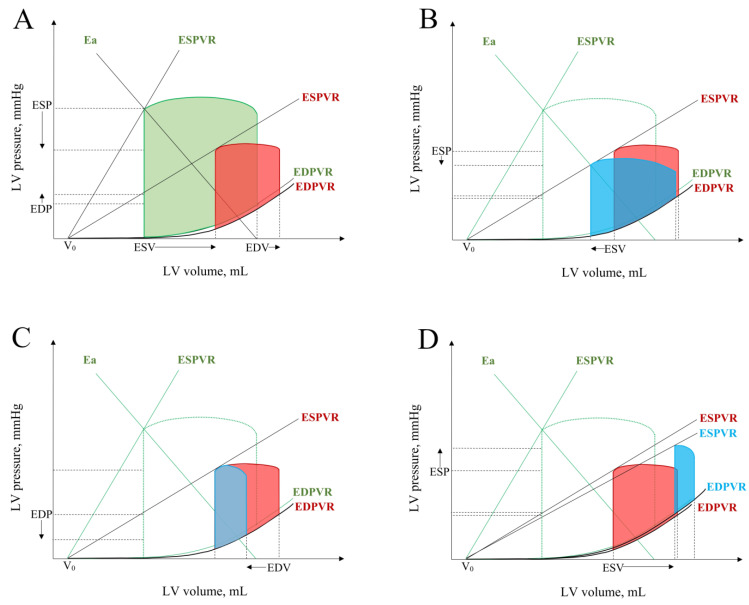
The response of LV pressure–volume loop in cardiogenic shock to short-term mechanical circulatory support. Cardiogenic shock reduces ESPVR slope and diminishes stroke volume (**A**). IABP reduces systolic blood pressure and increases stroke volume (**B**). Impella unloads the left ventricle by reducing its end-diastolic volume and pressure (**C**). ECMO covers peripheral blood demand without unloading of LV end-diastolic function (**D**). Abbreviations: IABP: intra-aortic balloon pump, ECMO: extracorporeal membrane oxygenation, LV: left ventricular, Ea: effective arterial elastance, EDV: end-diastolic volume, ESV: end-systolic volume, EDP: end-diastolic pressure, ESP: end-systolic pressure, ESPVR: end-systolic pressure-volume relationship, EDPVR: end-diastolic pressure-volume relationship. Green LV pressure–volume loop is for physiological conditions, red for cardiogenic shock, and blue for various types of short-term MCS.

The use of short-term MCS may be considered first in patients with potentially reversible CS. In the recommendations from 2005 [79], it was proposed to consider the MCS implantation if the chance of patient survival is more than 50%. In contrast, when the survival chance is lower than 10%, or the risk of disability is higher than 30%, as well as in patients with terminal disease, irreversible neurological damage, or multiple organ failure, MCS should not be considered. Anatomical contraindications for MCS include aortic dissection or severe aortic valve insufficiency. All available MCS including passive IABP, and active ECMO, TandemHeart or Impella in CS can be considered as a bridge-to-decision (BTD), bridge-to-recovery (BTR), bridge-to-bridge (BTB) (class IIaC) [12]. The various types of short-term MSC unload the left ventricle, prevent enhanced myocardial damage or provide optimal conditions for LV recovery (Figure 4).

The most frequently used system for mechanical circulatory support since the 1980s is IABP. A balloon inflated during the diastolic phase in the descending aorta increases the cardiac output by approximately 0.5 L/min. The meta-analysis of 7 randomized trials involving 1009 patients [80] showed that IABP as a supportive therapy during MI complicated by CS improves neither survival after 30 days nor LV ejection fraction but is associated with a higher incidence of stroke (by 2%, *p* = 0.03) or bleeding complications (by 6%, *p* = 0.02). On the other hand, the analysis of nine cohort studies showed that IABP was associated with an 18% (*p* < 0.001) lower 30-day mortality in MI patients treated with fibrinolytics but a 6% (*p* < 0.001) higher mortality in patients who underwent PCI.

The clinical significance of IABP in the era of modern mechanical reperfusion has been verified in the IABP-SHOCK II trial [81]. The trial results indicate that the addition of IABP to primary angioplasty in the treatment of peri-infarct CS did not reduce the 30-day mortality rate (39.7% vs. 41.3%, *p* = 0.69). Thus, the recent ESC guidelines do not recommend the routine use of IABP in patients with CS but IABP may be considered as a BTR, BTD, or BTB, in the case of mechanical complications (class IIbC) [12].

In patients with CS veno-atrial ECMO provides support for the cardio-pulmonary system pumping > 4 L/min of blood from the central veins or the right atrium through an oxygenator and a heat exchanger into the femoral or iliac arteries. The use of ECMO requires left-ventricle venting due to the lack of direct LV unloading and the time of its implantation should not exceed 14 days.

A clinical experience with ECMO in CS is derived from small observational studies. Out of 27 patients with CS on ECMO [82], 22 (81.5%) were weaned and 16 (59.3%) were discharged. Interestingly, 21 of them were resuscitated before ECMO implantation and the time of resuscitation and lactate concentration influenced clinical outcomes. In turn, in 134 patients with refractory CS of different etiology including MI, myocarditis or acute heart failure following cardiac surgery and with a mean initial systolic pressure of 50 mmHg maintained on multiple catecholamine infusion, ECMO allowed to wean 50.7% of patients and 42.5% of them were discharged. The use of ECMO in patients with refractory CS following MI with systolic blood pressure below 75 mmHg on inotropic drugs and IABP after primary coronary angioplasty was associated with a significant reduction in the 30-day mortality rate, from 68% to 33%, and 1-year mortality rate, from 76% to 36% [83]. A prospective, adequately powered, multicenter and randomized ECLS-SHOCK trial will address questions of efficacy and safety of ECMO in addition to early revascularization in acute MI complicated by CS [84]. Further research should be addressed to identifying patients at risk of developing left ventricular distension and pulmonary edema and how to optimally unload or vent the left ventricle during ECMO therapy. 

TandemHeart is a centrifugal pump that gives maximum cardiac output of 4 L/min. This device pumps venous blood from the left atrium approached by transseptal puncture into the abdominal aorta. The time required for its implantation is longer than that necessary for ECMO or Impella. In small, randomized studies it was shown that Tandem Heart significantly improved hemodynamic parameters as compared with IABP, however it did not influence the 30-day mortality rate [85]. In addition, Thiele et al. found a higher incidence of severe bleeding complications (90% vs. 40%, *p* = 0.002) and lower limb ischemia (33% vs. 0%, *p* = 0.009) with TandemHeart as compared to IABP [86]. 

Another available system for short-term MCS is the axial micropump, Impella, with a cardiac output of 2.5–5 L/min. Implanted through the aortic valve it pumps blood from the left ventricle into the ascending aorta. Its safety and effectiveness in the treatment of CS patients is derived from small clinical trials. In the ISAR-SHOCK trial, 26 patients with CS were randomly assigned to treatment with Impella 2.5 or IABP [87]. As in the TandemHeart system, Impella significantly improved cardiac output and mean arterial pressure, but had no effect on mortality after one month. In contrast to Tandem Heart, an Impella implantation was not associated with an increase in the incidence of lower limb ischemia as compared to IABP (8% vs. 0%). The retrospective analysis showed that, in patients with profound CS, those who were implanted with the Impella 5.0 system or changed from the Impella 2.5 pump to 5.0 more frequently survived the first month than patients in the group with the Impella 2.5 [88]. The results of Impella-EUROSHOCK registry showed [89] that the Impella 2.5 implantation in 120 CS patients was associated with a significant reduction of plasma lactate concentration, nevertheless the 30-day mortality rate remained high and amounted to 64.2%. In the recent IMPRESS-in-Severe-SHOCK trial, 48 patients with CS requiring mechanical ventilation were randomized to Impella CP versus IABP [72]. At 30 days, mortality in patients treated with either IABP or Impella CP was 50% and 46%, respectively. Moreover, at 6 months, the mortality rates for both Impella CP and IABP were 50%. There was also a lack of benefit in any of the other parameters, including arterial lactate. The safety and efficacy of Impella CP in MI patients with CS is currently being tested in the multicenter DanGer Shock trial. The primary endpoint of this study is 6-month all-cause mortality and a total of 360 patients are planned to be enrolled. 

Schrage et al. [70] have analyzed the data of 441,696 CS patients treated in German hospitals between 2005 and 2017. Although, the incidence of CS increased from 33.1/100,000 population in 2005 to 51.7/100,000 population in 2017, the proportion of CS due to acute MI decreased from 52.9% in 2005–2007 to 44.2% in 2014–2017. A high usage of IABP was found until 2010, while a growing use of ECMO and percutaneous left ventricular assist devices was observed thereafter. Over time, mortality remained high, at around 55–60%, with a slightly decreasing temporal trend in patients with acute MI. The ongoing and future MCS trials will have to clarify numerous issues associated with patient selection for MCS, the optimal type and timing of MCS implant, as well as issues related to weaning from MCS and the optimal approach to prevent and manage potential MCS-related complications. Additionally, new devices with fewer complications are expected.

## 8. Alternative Techniques Dedicated for Mechanical Complications

**Figure 5:**
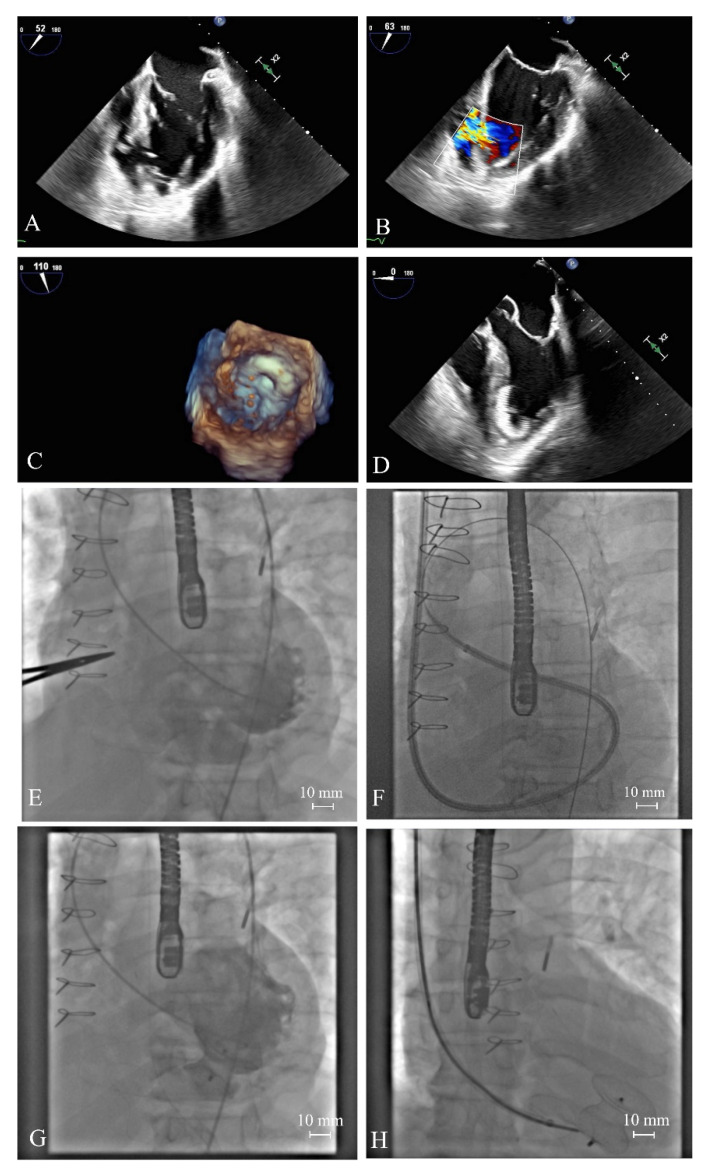
A patient with recanalized ventricular septal rupture initially treated with cardiac surgery. Ventricular septal rupture in transesophageal echocardiography (**A**,**B**) and LV angiography (**E**) treated with the Amplatzer occluder, inserted via the arteriovenous loop (**F**,**G**) with a nice effect (**D**,**H**). The LV surface of the Amplatzer occluder (**C**). LV: left ventricular.

Endovascular or hybrid procedures of VSR closure are usually performed under general anesthesia. A contraindication for the percutaneous closure of VSR is the size of rupture being greater than 35 mm, apical VSR location without a sufficient surrounding rim and location near the mitral, tricuspid and/or aortic valve. An occluder device is inserted via the arteriovenous loop into the region of interest in the interventricular septum and the procedure is navigated by echocardiography. When positioned correctly, one side of the implant remains in the right chamber while the other resides in the left chamber, sandwiching the defect [90] (Figure 5, Videos S2 and S3).

The endovascular techniques applied for VSR closure have some limitations. The stiff wire-based systems for the device delivery into the region of interest in the interventricular septum can cause disruption of the necrotic zone and increase the rupture size. Therefore, hybrid producers with alternative access via right ventricle puncture are used. The available occluders, anchored in fragile necrotic tissues of the septum, are often dislocated into the right ventricle, which usually requires cardiac intervention. The available sizes of device occluders may not be sufficient to close large defects and the residual left-to-right shunt remains one of the most common problems after such treatment. Thiele et al. [91] reported the results of treating postinfarction VSR using the Amplatzer occluder for an atrial or interventricular septal defect. Procedure-related complications such as major residual shunting, left ventricular rupture or device embolization occurred in 41%. Moreover, despite the successful implantation of the cardiac occluder in CS, 86% of them died within one month. Calvert et al. [92] have presented multicenter experience with catheter-based VSR treatment in 53 patients, including 19 after surgical closure. The shunt was completely or partially reduced in 85% of patients and 58% of patients survived to discharge and did well in the longer term.

Recently, first reports of acute MR following PMR successfully treated with a MitraClip have been presented [93,94]. In both cases, the experimental treatment was a rescue procedure for patients deemed too high risk for surgical intervention.

Percutaneous intrapericardial fibrin-glue injection therapy is an alternative therapy for FWR surgery, especially in an oozing FWR type [45]. Hattori et al. showed that fibrin glue, inserted into the pericardium, formed fibrin within 1 day, while the glue has degraded within a week [95]. Murata et al. in the autopsy following fibrin-glue injection did not find inflammatory adhesion of the epicardium to the pericardium [96]. Both studies indicate that fibrin glue is biocompatible and biodegradable.

Subsequent research on mechanical complications will be necessary to determine the optimal time window for VSR surgery, the role of MCS, and the development of new devices dedicated particularly for VSR and PMR. In case of mechanical complications, logistic issues concerning prehospital health care system and critical care training of the paramedics and physicians remain especially important.

## 9. The Concept of Personalized Therapy of Patients with Complicating MI 

The extremely high mortality rate in patients with complicating MI, one that remains at the level of 40–60% for many years, and has not been substantially reduced despite the use of new drugs and very advanced and expensive systems for cardiovascular support, provokes a reflection on a complete change of the approach to CS. From a medical point of view, the results of treatment of both severe and acute, systolic LV dysfunction as well as LV structural damage, brings frustration and discouragement, as it is caused by a localized and—it would seem—simple, mechanical reason that, if treated in the right time frame, has a huge potential to be reversed. On the other hand, for scientists it has become an inspiration for searching for new solutions.

The concept of personalized therapy of a patient with CS requires, first of all, an understanding of the specificity of the disease of each patient separately, recognizing the complexity of its clinical condition with the entire spectrum of surrounding circumstances, and finally selecting the most effective possible therapy. Currently, the specificity of each CS patient is determined by laboratory tests, echocardiography parameters and hemodynamic measurements [97,98,99,100,101]. In the future, these parameters may turn out to be insufficient, and the selection of the most effective therapy may involve the use of molecular biology techniques to profile the patient at the levels of genome, transcriptome, proteome and metabolome [102]. For this purpose, large prospective clinical trials will be necessary; based on which the first bio-catalogs of patients’ profiles will be collected. Afterwards, the most effective treatment methods associated with specific profiles will be identified. These, in turn, will be tested in well-controlled, prospective clinical trials.

Simultaneously, the multilevel profiling of patients has to be carried out along with research on innovative and miniaturized solutions, ones free from the complications of the previous generations of devices and avoiding the weaknesses of currently available therapeutic methods while providing flexibility in personalizing the invasive procedures. To be more effective in the future, we should draw more conclusions from the characteristics of patients who did not benefit from currently available treatment and devote the most creative fervor to them. Innovative, forthcoming methods should be tailored to patients who are dying, today, from complicating MI. 

We are convinced that the effective management of complicating MI including peri-infarct CS is possible. Therefore, the initiators and the editors of JCM have dedicated a special issue to this problem that can constitute a platform to share their own experience, reflections and ideas that have emerged while working with complicated MI patients.

## Data Availability

Not applicable.

## Supplementary Materials

The following are available online at https://www.mdpi.com/article/10.3390/jcm10245904/s1, Video S1: A patient with blowout type of free wall rupture. Coronary angiography revealed an acute ostial occlusion of left anterior descending artery (A). After coronary angiography, due to cardiac arrest following cardiac tamponade pericardiocentesis was performed. The catheter position was confirmed by contrast injection (B). Patient required cardiac surgery during which a blowout type of anterior wall rupture was found and sutured (C), Video S2: A patient with recanalized ventricular septal rupture initially treated with cardiac surgery in transesophageal echocardiography (A,B) and LV angiography (C). The Amplatzer occluder was inserted via the arteriovenous loop (D), Video S3: The treatment procedure of a patient from Video S2. Optimal position of a device assessed by echocardiography (A) and angiography (C). The left ventricular surface of the Amplatzer occluder (B,D).
